# Respiratory syncytial virus (RSV) antibody and small-molecule drugs: current status of clinical translation and challenges

**DOI:** 10.3389/fmicb.2026.1745808

**Published:** 2026-02-10

**Authors:** Hanxiang Liu, Hui Zhong, Hanmin Liu

**Affiliations:** 1 Department of Pediatric Pulmonology and Immunology, West China Second University Hospital, Sichuan University, Chengdu, China; 2 Key Laboratory of Birth Defects and Related Diseases of Women and Children, Ministry of Education, Sichuan University, Chengdu, China; 3 NHC Key Laboratory of Chronobiology, Sichuan University, Chengdu, China; 4 Department of Pediatric Pulmonology and Immunology, WCSUH-Tianfu·Sichuan Provincial Children's Hospital, Sichuan University, Meishan, China; 5 The Joint Laboratory for Lung Development and Related Diseases, West China Second University Hospital, Sichuan University, Chengdu, China; 6 School of Life Sciences, Fudan University, Shanghai, China; 7 West China Institute of Women and Children's Health, West China Second University Hospital, Sichuan University, Chengdu, China; 8 Sichuan Birth Defects Clinical Research Center, West China Second University Hospital, Sichuan University, Chengdu, China; 9 Department of Anesthesiology, Chengdu Third People's Hospital, Chengdu, China

**Keywords:** antiviral resistance, clinical translation, monoclonal antibodies, RSV, small-molecule antivirals

## Abstract

**Background:**

respiratory syncytial virus (RSV) remains a leading cause of lower respiratory tract infections worldwide, imposing a substantial disease burden on infants, older adults, and immunocompromised individuals. Despite its prevalence, therapeutic options have historically been limited, with no specific antiviral drugs widely approved for treatment until recently. The landscape is now shifting rapidly with the development of novel preventive and therapeutic agents.

**Content:**

this review comprehensively summarizes the current status of RSV monoclonal antibodies and small-molecule antivirals, integrating mechanistic insights with clinical translational perspectives. We analyze the evolution of immunoprophylaxis from palivizumab to next-generation long-acting antibodies like nirsevimab, which have reshaped prevention strategies. Furthermore, we evaluate small-molecule agents, contrasting the limitations of early fusion inhibitors with the improved efficacy and resistance barriers of emerging polymerase inhibitors such as ziresovir.

**Key issues:**

clinical translation faces multifaceted challenges beyond molecular discovery. Major hurdles include the complexity of clinical trial designs for vulnerable populations (neonates and the elderly), the lack of globally harmonized clinical efficacy endpoints, and the risks associated with viral escape mutations. Additionally, divergent regulatory frameworks and requirements across different regions complicate the global development and registration of new RSV products.

**Outlook:**

future advancements will likely depend on integrating emerging technologies, including mRNA platforms, gene editing, and AI-driven drug discovery. Moving forward, the field must prioritize multi-target combination therapies to mitigate resistance and establish global surveillance networks. Ultimately, international collaboration is essential to ensure equitable access, sustainable pricing, and the successful implementation of next-generation RSV therapeutics.

## 1 Introduction: global disease burden and unmet medical needs of respiratory syncytial virus (RSV)

Respiratory syncytial virus (RSV) is a single-stranded, negative-sense RNA virus belonging to the genus *Orthopneumovirus* in the family Pneumoviridae (Chen et al., 2023). It is one of the most common pathogens causing respiratory tract infections worldwide (Tongyoo et al., 2023). RSV infection exhibits diverse epidemiological characteristics, including sporadic outbreaks, localized epidemics, and seasonal global circulation. Together, these patterns impose substantial health and economic burdens, particularly among vulnerable populations (Mandal et al., 1985; Chadha et al., 2020).

RSV can be classified into two major antigenic subtypes, RSV-A and RSV-B, based on genetic and antigenic variability in the surface fusion (F) and attachment (G) glycoproteins (Hanage and Schaffner, 2025). These two subtypes share ~50% sequence homology, with the G protein exhibiting the highest degree of diversity, driving antigenic drift and immune evasion (Tan et al., 2013). Globally, both subtypes co-circulate each season, often displaying alternating predominance across years and regions (Yu et al., 2021). Some studies suggest that RSV-A infections are associated with more severe lower respiratory tract disease compared with RSV-B, although this finding remains debated (Nuttens et al., 2024). The genetic diversity and frequent emergence of novel genotypes within both subtypes pose substantial challenges for vaccine design and the development of broadly neutralizing antibodies (Nuttens et al., 2024). Consequently, a deeper understanding of RSV subtype molecular epidemiology is critical for the development of effective and broadly protective therapeutic and preventive strategies.

RSV is one of the primary causes of seasonal lower respiratory tract infections (LRTIs) in infants and remains one of the leading causes of virus-related neonatal mortality (Broadbent et al., 2015; Nicolas De Lamballerie et al., 2021). Globally, ~3.4 million children are hospitalized annually due to RSV infection, leading to around 200,000 deaths (Rajan et al., 2021). Notably, more than 90% of children are infected by RSV before the age of two, with infection peaks occurring between one and 6 months of age—making neonates and infants particularly vulnerable (De Ocana Sentuary et al., 2025; Hall et al., 2013).

Beyond pediatric populations, older adults and individuals with comorbidities also experience disproportionate disease severity (Sandrock and Stollenwerk, 2008). Patients with chronic obstructive pulmonary disease (COPD), asthma, or other underlying cardiopulmonary disorders are more susceptible to severe RSV infections. These infections are often associated with complications such as cardiovascular symptoms (Wang S. et al., 2025). Immunocompromised individuals, including hematopoietic stem cell or lung transplant recipients, also face prolonged viral shedding and heightened risk of severe disease progression (Shah and Chemaly, 2011).

Despite the immense global disease burden, there are still no effective antiviral drugs specifically approved for RSV treatment (Van Royen et al., 2022). Current therapeutic options, including ribavirin and palivizumab, have considerable limitations in efficacy, cost, and safety (Mejias and Ramilo, 2015). Palivizumab, the first preventive monoclonal antibody, requires multiple injections and high costs, affecting its widespread use (McGirr et al., 2017). Furthermore, the emergence of resistant viral strains has been reported (Saito et al., 2021). Recognizing these limitations, the World Health Organization has prioritized RSV vaccine and monoclonal antibody development within its Vaccine Product and Delivery Research agenda (Jesudason, 2025).

Driven by these challenges, worldwide pharmaceutical efforts are accelerating the clinical development of both RSV antibodies and small-molecule antiviral agents. The results of market analyses suggest an exponential growth trajectory for RSV therapeutics (Citron et al., 2024). According to the Fortune Business Insights report (2021), the global market for RSV therapeutics was valued at USD 609 million in 2020. It is projected to reach USD 4.0 billion by 2027, corresponding to a compound annual growth rate of ~30%.

**Figure 1:**
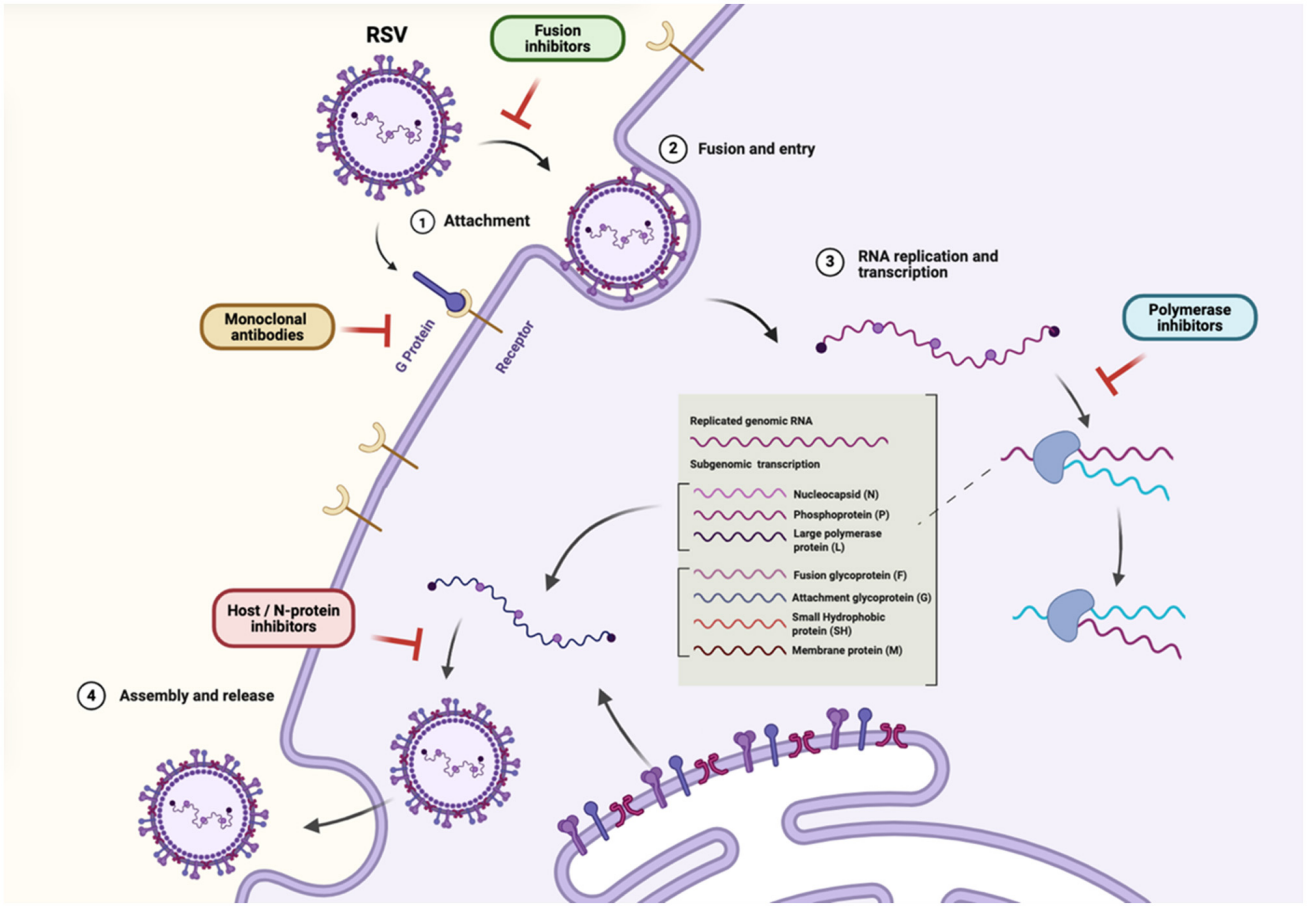
Schematic overview of the RSV life cycle and therapeutic intervention points.

To facilitate a clearer understanding of RSV's replication biology and therapeutic intervention points, Figure 1 provides a schematic overview of the RSV life cycle, highlighting the key stages of viral attachment, fusion, replication, and assembly. The figure also summarizes the molecular targets of currently available and emerging antiviral strategies. These include monoclonal antibodies that block viral entry and small-molecule inhibitors targeting the polymerase, nucleoprotein, or host-dependent replication processes.

This review provides a comprehensive summary of current advances in RSV antibody and small-molecule therapeutics, critically examines translational challenges, and discusses emerging strategies that may contribute to development of the next generation of RSV prevention and treatment. Distinct from existing RSV therapeutic reviews that primarily focus on either vaccine development, antibody prophylaxis, or antiviral pipelines, this review integrates mechanistic insights with translational, regulatory, and real-world perspectives. By systematically comparing antibody-based prevention and small-molecule therapeutics across clinical endpoints, regulatory expectations, and resistance risks, we aim to provide a unified framework to inform next-generation RSV drug development and implementation.

## 2 RSV monoclonal antibodies: from proof-of-concept to long-acting prophylaxis

Monoclonal antibodies currently constitute the most mature and clinically advanced strategy for RSV prevention. This section reviews the evolution of antibody-based prophylaxis from early proof-of-concept agents to next-generation long-acting monoclonal antibodies, with emphasis on translational success, remaining limitations, and lessons that have shaped current preventive paradigms.

### 2.1 Palivizumab: establishing passive immunoprophylaxis

Palivizumab was the first monoclonal antibody approved for the prevention of RSV infection and established the clinical feasibility of passive immunoprophylaxis in high-risk pediatric populations (The IMpact-RSV Study Group, 1998). By targeting the RSV fusion (F) protein, palivizumab demonstrated that antibody-mediated neutralization could reduce RSV-associated hospitalization in premature infants and those with underlying cardiopulmonary conditions (Feltes et al., 2003).

Despite this milestone, the clinical impact of palivizumab has been limited by several factors. Its requirement for monthly intramuscular administration during the RSV season, high acquisition cost, and restriction to narrowly defined high-risk groups constrained its scalability and population-level effectiveness (Michael et al., 2014). In addition, resistance-associated substitutions in the F protein were reported in treated patients, underscoring the vulnerability of first-generation antibodies to viral escape (Zhu et al., 2011).

Collectively, palivizumab provided critical proof-of-concept for RSV prevention while simultaneously highlighting the need for antibodies with extended half-lives, broader population applicability, and higher barriers to resistance—insights that directly informed the design of next-generation long-acting monoclonal antibodies (Mazur et al., 2023).

### 2.2 Nirsevimab: redefining RSV prevention through long-acting passive immunity

Nirsevimab represents a major advance in RSV prevention and marks a paradigm shift from high-risk–restricted prophylaxis to broad population-level protection. Unlike first-generation antibodies, nirsevimab targets a highly conserved epitope on the prefusion conformation of the RSV fusion (F) protein and incorporates Fc modifications that markedly extend serum half-life (Mazur et al., 2023; Zhu et al., 2017). This design enables a single intramuscular dose to provide protection across an entire RSV season.

Clinically, nirsevimab demonstrated consistent reductions in RSV-associated medically attended lower respiratory tract infection and hospitalization in both preterm and term infants, supporting its use beyond narrowly defined high-risk groups (Griffin et al., 2020). Importantly, its development program expanded the target population to include all infants entering their first RSV season, thereby reshaping clinical expectations for RSV prevention and aligning passive immunization strategies more closely with public health objectives (Hammitt et al., 2022).

From a translational and regulatory perspective, nirsevimab also redefined evidentiary standards for RSV prophylaxis. Its clinical trials supported streamlined dosing, simplified administration, and favorable safety profiles, facilitating regulatory approval pathways across multiple regions. At the same time, ongoing surveillance has identified rare escape-associated substitutions in the F protein, underscoring the continued need for resistance monitoring even for broadly neutralizing antibodies (Hammitt et al., 2022; Simões et al., 2023).

Collectively, nirsevimab established long-acting monoclonal antibodies as a viable, scalable approach to RSV prevention and set the benchmark against which subsequent next-generation antibodies are now evaluated (Mazur et al., 2023).

### 2.3 Next-generation long-acting monoclonal antibodies: incremental advances and remaining challenges

Building on the success of nirsevimab, several next-generation long-acting monoclonal antibodies targeting the RSV prefusion F protein have advanced into late-stage clinical development. These candidates are designed to further optimize dosing convenience, manufacturability, or breadth of neutralization while largely maintaining the same preventive paradigm established by nirsevimab (Syed, 2025).

Representative examples include clesrovimab and other regionally developed antibodies, which incorporate variations in epitope targeting or Fc engineering to enhance pharmacokinetic properties. To date, however, available clinical data suggest that these agents primarily offer incremental improvements rather than transformative gains in efficacy or population coverage (Syed, 2025). An overview of approved and late-stage monoclonal antibodies targeting RSV, including their mechanisms, clinical status, and target populations, is summarized in Table 1.

**Table 1:** Approved and late-stage monoclonal antibodies targeting RSV.

**Antibody**	**Target/ epitope**	**Indicated/ trial population**	**Dose and route**	**Pivotal trial(s)**	**Key efficacy outcomes**	**Safety profile**	**Regulatory status (2025)**
Palivizumab (The IMpact-RSV Study Group, 1998)	Site II (post-fusion)	High-risk preterm infants; CHD/CLD patients	15 mg/kg IM monthly × 5	IMpact-RSV (NEJM 1998)	↓ RSV-related hospitalization 55% vs. placebo	Generally well tolerated; mild injection site reactions	FDA 1998; EMA 1999 (approved for high-risk infants only)
Nirsevimab (Hammitt et al., 2022; Drysdale et al., 2023; Jorgensen, 2023)	Site Ø (prefusion)	All infants (first RSV season)	Single IM dose (50/100 mg)	Melody, harmonie, medley	↓ MA-LRTI ~75%; ↓ hospitalization ~83%	Comparable to palivizumab	Approved EU (2022), US (2023), CA (2023), JP (2024), CN (2024)
Clesrovimab (Syed, 2025)	Prefusion F (conserved epitope, Fc engineered for half-life extension)	Infants (first RSV season)	Single fixed dose IM	MK-1654-004/007	Met primary endpoints (MA-LRTI ↓ vs. placebo)	Similar to nirsevimab safety	Phase III completed; regulatory submission under review
TNM001	F protein (epitope undisclosed)	Infants ≤ 24 months	IM (single season)	Phase III ongoing (NCT pending)	Preliminary immunogenicity positive	Data pending	China phase III
RB0026	F protein (site II/IV region)	Infants (first season)	IM	Phase III initiated (2024)	Ongoing	Ongoing	China phase III
RV11	Sites IV–V (prefusion F)	Infants (prevention)	IM	Preclinical → Phase I	6.3 × greater lung protection vs. palivizumab in mice	Favorable animal tolerability	IND approved by China NMPA (2024)
REGN-RSV06	F protein (site V optimized)	Infants (prevention)	IM	Phase I–II ongoing	Data not yet public	–	Development ongoing
AK0610	F protein (prefusion)	Adults (phase I); planned infants	IM	NCT06996704	Favorable PK & safety in healthy adults	Mild AEs only	Phase I completed
RI-001/RI-002	Polyclonal RSV IgG	Immunocompromised patients	IV	Phase II	↓ bronchiolitis obliterans risk (trend)	Good tolerance	RI-002 licensed (for PID) – not RSV-specific
ALX-0171	F protein nanobody (trivalent)	Hospitalized infants	Inhaled nebulization	Phase IIb	Safety OK; antiviral endpoints not met	Mild AEs	Discontinued

CHD, congenital heart disease; CLD, chronic lung disease; MA-LRTI, medically attended lower respiratory tract infection; AE, adverse event; PK, pharmacokinetic; IM, intramuscular; IV, intravenous.

Importantly, the emergence of rare F-protein escape-associated substitutions across different antibody programs highlights a shared vulnerability of single-epitope targeting strategies (Sinha et al., 2025; Wetzke et al., 2025; Stobbelaar et al., 2025). Collectively, these observations underscore that while next-generation antibodies may refine existing approaches, achieving durable, population-wide RSV prevention will likely require continued surveillance and, potentially, complementary strategies beyond monoclonal antibodies alone.

This table summarizes approved and late-stage monoclonal antibodies to provide context for the evolution of antibody-based RSV prophylaxis, rather than a comprehensive inventory of all antibody programs. Data were compiled from pivotal clinical trials, regulatory submissions (FDA, EMA, PMDA, NMPA), and peer-reviewed or company-reported sources available up to October 2025. Arrows indicate direction of change: **↓**, decrease; **—**, not reported.

## 3 Small-molecule antivirals against RSV: translational lessons from successes and failures

Small-molecule antivirals represent a complementary strategy to antibody-based prophylaxis by enabling therapeutic intervention during active RSV infection. Unlike monoclonal antibodies, which are primarily used for prevention, small-molecule agents aim to suppress viral replication and modify disease progression after infection has occurred. Representative small-molecule antivirals and their key translational outcomes are summarized in Table 2.

**Table 2:** Representative small-molecule antivirals targeting RSV and key translational lessons.

**Compound**	**Molecular target**	**Clinical outcome**	**Key translational lesson**
Presatovir (Marty et al., 2020)	F protein (fusion)	Failed to improve outcomes in hospitalized adults/transplant patients	Efficacy is timing-dependent; entry inhibition may be insufficient in adults with established severe disease
Ziresovir (Zhao et al., 2024)	F protein (fusion)	Positive phase III efficacy signal (hospitalized infants)	Potent entry inhibition IS effective in infants with bronchiolitis, validating the fusion target in pediatric populations.
EDP-323 (Elmore et al., 2025)	L protein (polymerase)	Promising late-stage clinical data	Blocking replication downstream of entry offers a distinct mechanism with potentially higher barrier to resistance.
Sisunatovir (RV521) (DeVincenzo et al., 2020)	F protein (fusion)	Reduced viral load but missed some clinical endpoints	Consistent viral load reduction confirms antiviral activity, but clinical benefit correlation remains complex.

### 3.1 Fusion inhibitors: clinical validation and lessons learned

Fusion inhibitors were among the earliest classes of RSV small-molecule antivirals to demonstrate potent *in vitro* activity. Presatovir (GS-5806), a representative compound in this class, showed significant reductions in viral load in human challenge models (DeVincenzo et al., 2014). However, it failed to demonstrate clinical benefit in hospitalized adults with established RSV disease, highlighting the narrow therapeutic window of fusion inhibitors in certain populations (Marty et al., 2020).

In contrast to this setback, the landscape of fusion inhibitors was revitalized by Ziresovir (AK0529). Unlike Presatovir, Ziresovir demonstrated positive Phase III clinical outcomes in hospitalized infants, significantly reducing signs and symptoms of bronchiolitis. This success validates the fusion inhibition mechanism, suggesting that efficacy may depend heavily on the target population (infants vs. adults) and the timing of intervention (Zhang et al., 2025; Zhao et al., 2024).

### 3.2 Polymerase (L protein) inhibitors: targeting viral replication

Small-molecule inhibitors targeting the RSV RNA-dependent RNA polymerase (L protein) represent a distinct class of antivirals that act downstream of viral entry. By blocking viral RNA synthesis, these agents aim to suppress viral replication even after infection is established inside the cell (Fearns and Deval, 2016; Brookes et al., 2018).

Theoretical advantages of this class include a potentially broader therapeutic window compared to entry inhibitors and a higher barrier to resistance. Novel agents such as EDP-323 are currently under development to leverage this strategy. EDP-323 is a potent L-protein inhibitor designed with favorable pharmacokinetic properties to support once-daily oral dosing (Elmore et al., 2025). While this class is earlier in clinical translation compared to fusion inhibitors, it represents a promising frontier for therapeutic intervention.

This table highlights representative small-molecule RSV antivirals selected to illustrate key translational successes and failures rather than providing an exhaustive pipeline overview.

## 4 Key challenges in the clinical translation of RSV drugs

Beyond molecular and clinical considerations, broader implementation challenges will ultimately determine the real-world impact of RSV therapeutics. During clinical translation, both RSV antibodies and small-molecule antivirals face multifaceted challenges, particularly in clinical trial design and resistance management, which directly influence development efficiency as well as product safety and efficacy.

### 4.1 Clinical trial design challenges

The unique pathophysiology and population heterogeneity of RSV infection—especially across age groups and comorbid conditions—render clinical trial design complex.

#### 4.1.1 Complexity in trials for special populations

1. **High-Risk Populations:**
  RSV drug development should prioritize high-risk populations. These include infants under 24 months of age, particularly those born preterm or living with congenital heart disease, chronic lung disease, or immune deficiency (Guarnieri et al., 2025). Elderly adults and patients with chronic pulmonary disorders such as cystic fibrosis are also highly vulnerable. Immunocompromised individuals, such as hematopoietic stem cell or lung transplant recipients, experience more severe and prolonged disease. These populations differ substantially in physiology and immune responses. As a result, clinical trials should adopt customized designs and outcome measures to capture safety and efficacy across subgroups (Rafferty et al., 2022; Centers for Disease Control Prevention, 2024a; Zamora et al., 2025).
2. **Pediatric Populations:**
  Trials in pediatric RSV prevention and treatment must balance ethical limits, sampling feasibility, and clinical relevance. Studies often begin with high-risk infants, such as those born prematurely or with serious comorbidities. However, current regulations do not require proof of efficacy only in these groups before broader inclusion. Modern preventive options, including nirsevimab and the recently approved clesrovimab, are now recommended for all infants during their first RSV season. Additional dosing is advised for selected high-risk toddlers (Centers for Disease Control Prevention, 2024a,b; Syed, 2025). Pharmacokinetic sampling in neonates should follow sparse or population-based strategies to minimize burden. These approaches can still yield meaningful data.
3. **Elderly Populations:**
  Older adults represent a key population for RSV prevention and treatment. Comorbidities, polypharmacy, and age-related immune decline (immunosenescence) can reduce responsiveness to vaccines and antivirals. Clinical trial designs for this group should consider geriatric pharmacology principles and evaluate immune function parameters to better capture safety and efficacy outcomes (Miller and Niewiesk, 2025). Sanofi is currently evaluating a trivalent RSV/hMPV/PIV3 combination vaccine in adults aged 60 years and older (ClinicalTrials.gov, NCT06604767).
4. **Immunocompromised Patients:**
  People with weakened immune systems, including post-transplant or immunosuppressed patients, face greater risks of severe disease and viral persistence. Studies in hematopoietic stem cell transplant recipients have documented viral persistence lasting several weeks to months (Hammitt et al., 2022). These findings underscore the need for careful clinical monitoring and infection-control strategies (Lehners et al., 2016). Reports in immunocompromised cohorts also highlight higher morbidity and mortality associated with RSV infection. Viral evolution has been observed during prolonged RSV infections. Ongoing genomic surveillance is therefore encouraged to better characterize resistance emergence and potential immune escape (Kim et al., 2022).

#### 4.1.2 Appropriate clinical endpoint selection

1. **Uncertainty of Endpoints:**
  At present, there is no universally accepted set of clinical efficacy endpoints for RSV prevention or treatment trials. Differences in patient age, disease severity, and clinical presentation complicate endpoint harmonization across studies. Regulatory authorities, including the U.S. Food and Drug Administration (FDA) and the European Medicines Agency (EMA), emphasize early dialogue between sponsors and regulators. Such discussions aim to define robust, reproducible, and clinically meaningful endpoints tailored to the target population and intervention type (US Food Drug Administration, 2019; European Medicines Agency, 2023).
2. **Clinical Outcomes as Primary Endpoints:**
  For pivotal Phase III trials of RSV prevention or treatment, primary endpoints should focus on clinically meaningful outcomes. These include hospitalization, ICU admission, requirement for mechanical ventilation, symptom severity scores, and mortality. These endpoints reflect direct patient benefit and align with regulatory expectations for demonstrating efficacy. Current guidance encourages the inclusion of both virologically confirmed RSV infection and clinically relevant outcomes—such as medically attended lower respiratory tract infection (MA-LRTI), hospitalization, and mortality—as primary efficacy measures (Walsh et al., 2023). In contrast, viral-load reductions and other surrogate measures may serve as exploratory or secondary endpoints. However, they are not yet universally accepted as primary efficacy measures (US Food Drug Administration, 2019; European Medicines Agency, 2023; Xing and Bahl, 2025).
3. **Exploration of Surrogate Endpoints:**
  Validated surrogate markers capable of reliably predicting clinical benefit in RSV prevention or treatment remain undefined. While reductions in viral load or improvements in pharmacodynamic (PD) parameters can help guide dose-ranging and proof-of-concept studies in Phase II, such measures are not yet accepted as primary efficacy endpoints for pivotal Phase III trials (US Food Drug Administration, 2019; Ma et al., 2025). Exploratory biomarkers—including viral load kinetics, serum neutralizing antibody titers, inflammatory cytokines, and host immune transcriptomic profiles—may provide insights into disease pathogenesis and intervention response. However, further validation is required before these markers can be integrated into regulatory decision-making or post-marketing surveillance frameworks (Ma et al., 2025; Deng et al., 2025).
4. **Immunogenicity Evaluation:**
  If vaccine efficacy is thought to depend primarily on cellular or mucosal immunity, early identification of meaningful immunogenicity metrics is advisable. Comparative immunogenicity assessments across study arms typically include geometric mean titers (GMTs), geometric mean concentration (GMC) ratios, seroconversion rates or positivity-rate differences. While such immunologic endpoints offer useful insights in early-phase trials, it remains critical to link them to clinical protection through further validation (Deng et al., 2025; Shaw et al., 2024; Pang et al., 2024).

#### 4.1.3 Ethical considerations, trial scale, and duration

1. **Ethical Considerations**
  Ethical principles are essential in RSV clinical research, especially for pediatric and vulnerable groups. International guidelines emphasize that **adult safety data should be collected before enrolling infants or children**. This step ensures that dosing and potential risks are better understood. Participants with known hypersensitivity to vaccine or immunoglobulin components should be excluded. Those who have received other RSV prophylactic products in the same season should also be excluded to avoid confounding. Informed consent must be comprehensive and include discussion of long-term safety and uncertainty about immune effects. Ethical committees are advised to review pediatric protocols in a staged manner. Enrollment should proceed in a staged manner. Studies may move from adults to older children and then to younger groups only after predefined safety milestones are met (European Medicines Agency, 2023; WHO, 2020).
2. **Trial Design and Control Selection**
  Both prophylactic and therapeutic studies should use **randomized, double-blind, controlled designs** whenever possible to reduce bias. When blinding is impractical, objective endpoint adjudication and independent monitoring should be applied. For prophylactic vaccine trials, placebo-controlled superiority designs are acceptable until a licensed product becomes available. After that, **active-controlled non-inferiority** designs are preferred. Therapeutic studies should use approved antivirals or recognized standard-of-care treatments as positive controls if placebo use would be unethical (US Food Drug Administration, 2019; European Medicines Agency, 2023).
3. **Risk Stratification and Population Selection**
  Randomization should take into account factors that influence RSV severity. These include gestational age, prematurity, congenital heart disease, chronic lung disease, and regional differences in RSV season. Novel delivery routes, such as inhaled or intranasal formulations, should first be tested in healthy adults. Evaluation in patients with pulmonary disease can follow once initial safety is confirmed (European Medicines Agency, 2023; WHO, 2020).
4. **Safety Databases and RWE**
  The size of the safety database for approval depends on the expected benefit–risk profile and preclinical toxicology data (US Food Drug Administration, 2019). The database should include participants who received proposed or higher doses long enough to detect uncommon adverse events. RWE can strengthen clinical findings. It supports post-marketing surveillance, label extension, and validation of safety biomarkers. Integrating RWE from registries, pharmacovigilance data, and health-record systems provides important long-term safety information (Prunas et al., 2024; Branche, 2024; Bollaerts et al., 2024).

#### 4.1.4 Regulatory guidance and registration pathways

1. **Scope and Early Engagement**
  Global regulatory authorities emphasize early scientific consultation. This approach helps ensure alignment on study design, primary endpoints, estimands, and statistical strategy before pivotal RSV prevention or treatment trials. Early engagement minimizes development risk, supports harmonization, and facilitates global data acceptance under ICH E6 (R3), E8 (R1), and E9 (R1) (International Council for Harmonisation of Technical Requirements for Pharmaceuticals for Human U, 2023, 2021a, 2019).
2. **Prevention vs. treatment products**
  - **Preventive          (Vaccines/Long-acting Monoclonal Antibodies):**
    - Primary endpoints should capture *clinically meaningful outcomes* such as RSV-confirmed MA-LRTI, hospitalization, ICU admission, or death, rather than purely virologic surrogates (US Food Drug Administration, 2023; Moulia et al., 2025; European Medicines Agency, 2013).
    - Immunologic measures (neutralizing antibody titers, seroconversion rates, mucosal immunity) can serve as supportive or bridging endpoints but must be correlated to protection.
    - Batch consistency, CMC comparability, and durable safety data are essential for licensure.
  - **Therapeutic (Antivirals/Therapeutic mAbs):**
    - Efficacy endpoints should focus on time to clinical recovery, disease progression, mechanical ventilation, or mortality.
    - Viral-load reduction may serve exploratory or secondary roles.
    - Monitoring for viral resistance and immune escape through genotyping/phenotyping is required, especially in immunocompromised patients.
3. **Special Populations**
  - **Pediatric:** Adult safety data should precede infant enrollment. Studies should apply ethical sampling (sparse PK, minimal blood volume) and may extrapolate from older populations if immunologic pathways are comparable (per ICH E11A) (International Council for Harmonisation of Technical Requirements for Pharmaceuticals for Human U, 2021b).
  - **Older Adults:** Designs must address immunosenescence, frailty, polypharmacy, and comorbidities, including immune-function biomarkers to interpret variable vaccine responses.
  - **Immunocompromised Patients:** Require prolonged follow-up, adaptive cohort designs, and extended safety surveillance to capture delayed or atypical responses.
4. **Endpoints, Estimands & Statistical Planning**
  Regulators encourage clear definition of estimands (target population, intervention, control, endpoint, handling of intercurrent events) (International Council for Harmonisation of Technical Requirements for Pharmaceuticals for Human U, 2019). Seasonal variation and regional differences should be handled by stratified randomization and pre-specified interaction analyses. Multiplicity and non-inferiority margins must be justified with historical data and clinical rationale.
5. **Resistance and Variant Monitoring**
  Continuous genomic surveillance and phenotypic testing are recommended for all RSV antivirals and antibodies to detect emerging resistance or antigenic drift. Lifecycle plans should anticipate strain updates, dose adjustments, and bridging immunogenicity studies similar to influenza and SARS-CoV-2 precedents (US Food Drug Administration, 2021).
6. **Safety Databases and RWE**
  The size of the safety database should reflect exposure and risk profile of the target population. Long-term RWE sources (e.g., registries, electronic health records, vaccine safety databases) are valuable for detecting rare events and confirming effectiveness.
7. **Accelerated and Conditional Approval Pathways**
  Regulators (FDA, EMA, MHRA, Health Canada, PMDA) offer mechanisms such as Fast Track, Breakthrough Therapy, Priority Review, PRIME, or Conditional Marketing Authorization for RSV products targeting unmet needs. Surrogate endpoints (e.g., immunologic correlates) may support such approvals if well-justified and confirmed in post-marketing studies (US Food Drug Administration, 2014; European Medicines Agency, 2020).
8. **Comparative overview of global regulatory focus**
  To facilitate global alignment, Table 3 summarizes the convergent regulatory priorities across major agencies. Although procedural details vary, most frameworks emphasize similar expectations for clinical endpoints, pediatric development, and pharmacovigilance systems.

**Table 3:** Comparative regulatory focus for RSV vaccine and therapeutic development.

**Dimension**	**FDA**	**EMA**	**WHO/ICH alignment**
Guiding documents	Guidance for industry: RSV prevention in infants and children (2023/2024)	Guideline on clinical evaluation for RSV disease (EMA 2019)	ICH E6 (R3), E8 (R1), E9 (R1), E11A
Primary efficacy endpoint	Clinically meaningful (hospitalization, MA-LRTI, death)	Same, with focus on RSV-confirmed MA-LRTI	Harmonized definitions across regions
Pediatric development	PREA/BPCA plans; adult → child staged approach	PIP mandatory; stepwise safety and efficacy	ICH E11/E11A for extrapolation
Elderly/frailty considerations	Immunosenescence and comorbidity evaluation	Similar; recommends immunologic sub-analyses	WHO SAGE principles
Accelerated pathways	Fast track/breakthrough/priority review/accelerated approval	PRIME/conditional marketing authorization	WHO emergency use listing (EUL)
Post-marketing safety	Risk management plan (RMP), VAERS, RWE	RMP/PASS required	ICH E2E pharmacovigilance consistency

#### 4.1.5 Practical regulatory differences and implications for RSV drug development

Although regulatory authorities share broadly aligned principles for the development of RSV preventive and therapeutic products, important differences in emphasis remain across regions, with direct implications for clinical trial design and translational strategy.

With respect to paediatric development, both the FDA and the EMA endorse a staged approach, typically progressing from adults to older children and subsequently to infants once adequate safety and pharmacokinetic data are available. The EMA, however, requires a formal Paediatric Investigation Plan (PIP), which necessitates early definition of paediatric study timelines and endpoints. In contrast, the FDA framework under PREA/BPCA places greater emphasis on early scientific consultation and risk-based flexibility, provided that benefit–risk considerations are well justified (European Medicines Agency, 2013; US Food Drug Administration, 2024). In China, regulatory expectations for paediatric RSV products continue to evolve. While increasing alignment with international standards is evident, early engagement with the National Medical Products Administration (NMPA) may be particularly valuable for products targeting neonates or very young infants.

Regarding clinical endpoints, regulators consistently prioritize clinically meaningful outcomes such as RSV-confirmed MA-LRTI, hospitalization, or disease progression. The EMA places particular emphasis on standardized RSV-confirmed MA-LRTI definitions, whereas the FDA allows more flexibility in endpoint hierarchies when supported by robust secondary analyses (European Medicines Agency, 2013; US Food Drug Administration, 2024). These differences highlight the importance of early endpoint harmonisation in multinational trials, including alignment on diagnostic criteria and assessment windows. Virologic endpoints, while informative in early-phase studies, are generally considered supportive rather than primary in late-stage development (Modjarrad et al., 2016).

Antiviral resistance is increasingly viewed as a lifecycle consideration. The FDA emphasizes longitudinal resistance monitoring and post-marketing surveillance, while the EMA typically integrates resistance evaluation into Risk Management Plans and post-authorization studies (European Medicines Agency, 2013; US Food Drug Administration, 2024). In China, resistance surveillance requirements are developing alongside expanding clinical use. Incorporation of genomic monitoring and resistance risk assessment into development and post-marketing plans may facilitate regulatory review and long-term clinical adoption.

### 4.2 Challenges of antiviral resistance

The emergence of resistant strains of RSV remains a critical barrier to effective antiviral therapies. RSV's high mutation rate and adaptive ability enable it to escape drug pressure, reducing the efficacy of current treatment options. This section examines the potential mechanisms underlying antiviral resistance. It also discusses strategies for monitoring resistant strains and emerging approaches to counteract resistance during clinical development.

#### 4.2.1 Mechanisms of resistance

Resistance to RSV antivirals can arise through various mechanisms, including mutations in viral proteins, adaptive replication strategies, and cellular drug-efflux mechanisms.

1. **Viral Protein Mutations**:
  The F and L proteins are key targets of RSV antiviral drugs. Mutations in the RSV F protein, particularly in regions recognized by monoclonal antibodies, can reduce antibody binding affinity. This reduction facilitates immune evasion. This has been observed with drugs like **palivizumab**, where immune escape mutations in the F protein (e.g., K272E/Q/M and N276S) have been documented. Similarly, mutations in the RSV L protein, targeted by polymerase inhibitors, can diminish drug efficacy (Yasui et al., 2016; Zhao and Song, 2024).
2. **Altered Replicative Mechanisms**:
  RSV may compensate for drug inhibition by modifying its replication mechanisms. For example, mutations in the viral polymerase or co-factors can enable the virus to replicate in the presence of antiviral agents, contributing to resistance (Kleiner et al., 2023).
3. **Efflux Mechanisms**:
  Cells infected with RSV may upregulate efflux pumps that actively expel antiviral drugs. This process can lower intracellular drug concentrations and reduce antiviral effectiveness. While this remains speculative for RSV, it is a target for future research (Li et al., 2025; Mahey et al., 2024).

#### 4.2.2 Global resistance surveillance strategies

Monitoring and addressing RSV resistance requires robust global surveillance systems. Several strategies can be employed to detect and mitigate the impact of resistant strains.

1. **Genomic Sequencing:**
  High-throughput sequencing technologies are invaluable in identifying resistance-associated mutations within viral genomes (WHO, 2025). This approach enables real-time tracking of viral evolution and can help identify emergent strains that may reduce the efficacy of current therapies (Holland et al., 2023).
2. **Phenotypic Resistance Testing:**
  Cell-based assays that evaluate the growth of resistant strains in the presence of antiviral drugs remain the gold standard for detecting phenotypic resistance. Although time-consuming, this method is crucial for confirming findings from genomic studies (Stobbelaar et al., 2025).
3. **Epidemiological Networks:**
  Surveillance networks, such as those operated by national health organizations (e.g., CDC), can monitor RSV resistance patterns across different regions. These global networks allow for early detection and response to shifts in viral strains (WHO, 2019).

#### 4.2.3 Approaches to overcoming or preventing resistance

To ensure the long-term effectiveness of RSV antivirals, several strategies should be integrated into drug development pipelines.

1. **Targeting Conserved Viral Epitopes**:
  Developing drugs that target conserved regions of the RSV F, L, or N proteins is one strategy to minimize the impact of resistance. For example, **RV11** binds to conserved sites on the F protein, reducing the risk of escape mutations (Dai L. et al., 2023; Sun et al., 2024a).
2. **Combination Therapies**:
  Combining antivirals with different mechanisms of action (e.g., a polymerase inhibitor with a fusion inhibitor) can enhance therapeutic efficacy and reduce the likelihood of resistance emergence. Drugs like **RV11** and **ziresovir** show promise when used in combination, potentially providing broad protection against diverse RSV strains (Song et al., 2024).
3. **Personalized Medicine**:
  The use of genomic sequencing to tailor antiviral therapies to specific resistance profiles could significantly improve treatment outcomes. This approach allows for more targeted therapy, particularly for immunocompromised individuals who may be at higher risk for prolonged RSV infections and resistance development (Song et al., 2024; Li et al., 2026).
4. **Novel Drug Classes**:
  Exploring new classes of drugs, such as PROTACs (proteolysis-targeting chimeras), which can degrade viral proteins, offers a novel way to tackle antiviral resistance. These drugs can target multiple steps of viral replication and potentially avoid resistance due to the virus's ability to mutate at specific binding sites (Dosbaa et al., 2024).

#### 4.2.4 The role of RWE

The integration of RWE plays a crucial role in monitoring the long-term effectiveness of RSV antivirals and identifying emerging resistance patterns. Post-marketing surveillance, including data from registries and health records, can provide valuable insights into the performance of RSV drugs in diverse populations. This information is essential for updating clinical guidelines and adjusting therapeutic strategies based on real-world resistance trends (Zhang et al., 2025; Branche, 2024).

## 5 Future trends and perspectives in RSV drug development

The future of RSV therapeutics will be shaped by advances in technology and multi-target treatment strategies. At the same time, there is increasing emphasis on achieving both precision and broad accessibility. Innovations in mRNA technology, gene editing, and next-generation antibody engineering are paving the way for more effective treatments and vaccines. This section discusses the promising strategies and opportunities for improving RSV prevention and treatment.

### 5.1 Emerging technologies in drug development

1. mRNA Technology:
  - **Advantages and Potential:** mRNA platforms offer unprecedented speed, safety, and flexibility in developing therapeutic solutions for both infectious diseases and rare conditions. mRNA-based RSV vaccines can encode complex intracellular targets. This capability may allow intervention against previously “undruggable” viral targets. For example, Sanofi's RSV mRNA vaccine, currently in Phase II, aims to evolve into a multivalent respiratory virus vaccine. Additionally, Deep Vaccine Biotech is working on a bivalent RSV mRNA vaccine, IN006, which is still in early development stages (Wilson et al., 2023; Terstappen et al., 2024).
  - **Delivery Challenges:** While lipid nanoparticles (LNPs) have proven effective as the primary delivery system for mRNA vaccines, challenges remain in terms of production, patent constraints, and optimizing LNP stability, efficiency, and safety. Research is ongoing to improve LNP formulations for broader clinical applications (Hariri et al., 2025; Jang et al., 2025).
2. Gene Editing:
  - **CRISPR/Cas9 Breakthrough:** The approval of CRISPR-based therapies for genetic disorders such as β-thalassemia and sickle cell disease mark a major milestone (Sheridan, 2024; Healey, 2024; Liu et al., 2025). CRISPR technology holds the potential to enhance host resistance or directly disrupt the RSV genome, providing an innovative approach to RSV therapy in the future.
3. Next-Generation Antibody Engineering:
  - **Antibody–Drug Conjugates (ADCs) and Bispecific Antibodies:** These advanced antibody designs allow for targeted drug delivery and dual-epitope binding, improving therapeutic efficacy while minimizing the development of resistance (Wang R. et al., 2025). ADCs offer a novel way to combine the strengths of traditional monoclonal antibodies with cytotoxic agents to target RSV-infected cells more effectively. Bispecific antibodies are engineered molecules capable of simultaneously binding two distinct epitopes, offering enhanced viral neutralization and reduced potential for resistance. Recent research has explored bispecific designs in RSV immunotherapy, including molecules that target RSV-neutralizing B-cell receptors to elicit broadly protective antibody responses (Scharffenberger et al., 2024).
  - **Long-Acting Antibodies**: The recent approval of long-acting antibodies like nirsevimab and clesrovimab reflects a paradigm shift towards extended protection for RSV. Ongoing research aims to extend the half-life and broaden the spectrum of these antibodies, improving patient convenience and coverage (Diethelm-Varela et al., 2023; Mullard, 2023).

### 5.2 Design principles for next-generation antibodies and small molecules

1. **Multi-Target Strategy:** Targeting multiple steps in the RSV replication cycle is expected to become the standard strategy. Combination therapies that integrate both monoclonal antibodies and small-molecule drugs will not only improve efficacy but also reduce the likelihood of antiviral resistance (Bonneux et al., 2024; Gao et al., 2021).
2. **Optimization of Oral Small Molecules:** Oral antivirals, such as ziresovir and ASC10, have proven benefits, particularly in pediatric care. Future development will focus on optimizing the bioavailability, pharmacokinetics, and safety profiles of these agents, making home-based treatments more convenient and effective (Zhao et al., 2024; Dai P. et al., 2023).
3. **Personalized Medicine Potential:** The integration of genomics, transcriptomics, and proteomics will enable precision medicine for RSV treatment. By conducting rapid genotyping, clinicians will be able to select the most effective drugs for patients based on specific resistant strains of RSV (Yang et al., 2025).

### 5.3 Global market landscape and opportunities in rsv drug development

The global landscape for RSV drug development is evolving rapidly, driven by technological advances and innovation across various regions. Leading biotech firms in North America and Europe, such as Sanofi, AstraZeneca, and Regeneron, are pioneering long-acting monoclonal antibodies like nirsevimab and clesrovimab, shaping global prevention strategies for high-risk populations (Terstappen et al., 2024; Jorgensen, 2023; Lee et al., 2025).

This diverse global effort underscores the importance of international collaboration to advance RSV therapeutics and ensure effective prevention and treatment world widely.

### 5.4 Potential solutions and forward-looking insights

1. **Pan-Viral Therapeutics:**
  Given the prevalence of co-infections (e.g., influenza, rhinovirus), broad-spectrum antiviral therapies targeting conserved viral structures or host factors could provide high clinical value. These pan-viral agents could be especially valuable in treating patients with multiple infections or when precise viral identification is not possible (Luong et al., 2025; Karim et al., 2023).
2. **AI-Driven Drug Discovery:**
  The application of artificial intelligence (AI) can significantly accelerate the identification of therapeutic targets, compound optimization, and resistance prediction. AI-driven tools could also provide critical insights into preemptive resistance management, improving the longevity of current and future antiviral drugs (Kandeel et al., 2024; Wu et al., 2024).
3. **Vaccine–Therapeutic Synergy:**
  A comprehensive approach to RSV prevention and treatment could combine vaccines and antivirals. The integration of preventive vaccines with therapeutic antivirals offers a robust strategy for high-risk populations, enhancing protection during both early infection and after exposure periods (Sun et al., 2024b).
4. **Novel Delivery Systems:**
  Advances in delivery technologies, such as polymer nanoparticles and extracellular vesicles, could overcome some of the challenges associated with LNPs, enhancing the stability and efficiency of mRNA vaccines and gene-editing tools (Deng et al., 2024).
5. **Global Surveillance Network:**
  Establishing a global surveillance network that integrates genomic sequencing and AI-powered analytics will enable real-time monitoring of RSV strain evolution. Such systems will also facilitate early detection of emerging antiviral resistance. This would help guide treatment strategies and ensure that the global response to RSV remains agile and informed.
6. **Economic Accessibility:**
  Ensuring the affordability and accessibility of RSV therapeutics world widely will be crucial. Sustainable pricing models, along with international cooperation, will help make these treatments available to populations across various economic settings (Hutton et al., 2024).

## 6 Conclusion and future direction

RSV remains a leading cause of lower respiratory tract infection in infants, older adults, and immunocompromised individuals. Although major progress has been made, RSV still poses important challenges in both prevention and treatment.

Long-acting monoclonal antibodies such as nirsevimab and clesrovimab provide single-dose, season-long protection and have transformed RSV prevention. Oral antivirals such as ziresovir and polymerase inhibitors like EDP-323 represent important advances in therapeutic development. However, clinical translation remains difficult. Trials in neonates and older adults are complicated. Clinical endpoints differ among studies, and real-world evidence is still limited. Resistance-related mutations, first seen with palivizumab and REGN2222, highlight the need for ongoing molecular surveillance and adaptive clinical strategies.

Future progress will depend on integrating new technologies with clinical and public health practice. mRNA vaccines, gene editing, and AI-assisted drug discovery may improve efficacy and shorten development time. Yet biological innovation alone is not enough. Wider access, regulatory harmonization, and sustainable pricing are essential to achieve population-level impact, especially in low- and middle-income countries. Strong real-world data and coordinated surveillance programs will be key to maintaining long-term effectiveness. Importantly, despite recent scientific advances, access to RSV monoclonal antibodies and antivirals remains highly uneven across regions. High manufacturing costs, cold-chain requirements, and limited reimbursement mechanisms pose substantial barriers in low- and middle-income countries, where the burden of RSV disease is often greatest. Addressing these disparities will require coordinated strategies, including tiered pricing, technology transfer, and alignment with global procurement initiatives.

In conclusion, RSV therapeutics are moving from discovery to implementation. Continued innovation, global collaboration, and attention to access and resistance will be crucial to achieve effective and lasting control of worldwide RSV infection associated diseases.
